# VarGuideAtlas: a repository of variant interpretation guidelines

**DOI:** 10.1093/database/baaf017

**Published:** 2025-03-11

**Authors:** Mireia Costa, Alberto García S., Oscar Pastor

**Affiliations:** PROS Research Center, VRAIN, Universitat Politècnica de València, Cami de Vera, S/N, Valencia, Valencia 46022, Spain; PROS Research Center, VRAIN, Universitat Politècnica de València, Cami de Vera, S/N, Valencia, Valencia 46022, Spain; PROS Research Center, VRAIN, Universitat Politècnica de València, Cami de Vera, S/N, Valencia, Valencia 46022, Spain

## Abstract

Variant interpretation guidelines guide the process of determining the role of DNA variants in patients’ health. Currently, hundreds of guidelines exist, each applicable to a particular clinical domain. However, they are scattered across multiple resources and scientific literature. To address this issue, we present VarGuideAtlas, a comprehensive repository of variant interpretation guidelines that compiles information from ClinGen, ClinVar, and PubMed. Our repository offers a user-friendly web interface with advanced search capabilities, enabling clinicians and researchers to efficiently find relevant guidelines tailored to specific genes, diseases, or variant types. We employ ontologies to characterize each guideline, ensuring consistency and improving interoperability with bioinformatics tools. VarGuideAtlas represents a significant advance toward standardizing variant interpretation practices, facilitating more informed decision-making, improved clinical outcomes, and more precise genomic research. VarGuideAtlas is publicly accessible via a web-based platform (https://genomics-hub.pros.dsic.upv.es:3016/).

## Introduction

Advancements in next-generation sequencing technologies have significantly accelerated the identification of DNA variants in a patient’s genome and reduced the cost of this process [1]. Building on these advancements, geneticists and researchers have increasingly focused on understanding the impact of genetic variants on human health and disease, a process known as variant interpretation. This complex process involves a comprehensive evaluation of multiple types of evidence associated with the variant under study, such as its prevalence in populations and previous expert evaluations of its clinical significance [2].

Despite advances in the identification of genetic variants, the interpretation of their clinical significance remains a significant challenge. The inherent complexity of variant interpretation has led to a process prone to subjectivity that lacks systematization. This results in considerable variability in how experts interpret variants and assess their pathogenicity, often differing between individuals and institutions. In an effort to address these issues and reduce subjectivity by providing more precise definitions, variant interpretation guidelines were conceived. These guidelines aim to establish standardized procedures for evaluating the available evidence and assessing the clinical significance of variants based on predefined criteria.

The American College of Medical Genetics and Genomics (ACMG) and the Association for Molecular Pathology (AMP) pioneered variant interpretation guidelines in 2015 [3]. These guidelines helped geneticists conduct more consistent and evidence-based interpretations of genetic variants. The ACMG–AMP 2015 guidelines quickly gained widespread adoption [4]. However, these guidelines were not a definitive solution due to their generic nature [5]. The guidelines themselves emphasize that they must be adapted by experts in order to accommodate the nuances of specific genes, diseases, and types of variants. This customization process, often referred to as “guideline adaptation” [6], is critical for ensuring the accuracy and relevance of variant interpretation in specific clinical contexts.

This adaptation process has resulted in hundreds of new guidelines tailored to specific fields or offering alternative approaches. While these specialized guidelines allow geneticists to conduct evidence-based and more consistent interpretations of genetic variants in particular contexts, their proliferation poses significant challenges. Clinicians and researchers now face the challenge of identifying the most suitable guideline for their specific needs among a big and growing number of options. This difficulty is further compounded by the lack of a centralized repository that hosts all available guidelines. Instead, they are scattered across various literature sources and web resources maintained by different organizations and laboratories, making it challenging to locate and compare them effectively.

Given this context of fragmentation, there is a critical need for a centralized repository that systematically collates and organizes these guidelines. This resource would enable users to quickly search and access them based on their specific requirements, ultimately improving the efficiency and consistency of variant interpretation.

This work presents VarGuideAtlas, a curated repository that compiles existing variant interpretation guidelines and offers a detailed, ontology-guided description of their applicability in specific contexts. VarGuideAtlas provides several key benefits, of which we highlight the following: first, it centralizes access to current variant interpretation guidelines, thereby supporting informed decision-making in genomic medicine. Second, it offers advanced search capabilities, allowing users to find relevant guidelines by disease, gene, variant type, or keyword, making their retrieval more efficient. Third, it helps researchers and clinicians identify the most relevant guidelines for specific clinical scenarios, potentially reducing the risk of interpretation errors and misapplication. Finally, VarGuideAtlas contributes to the standardization of variant interpretation practices by facilitating access to established guidelines, ultimately improving the consistency and reliability of variant interpretation.

## Materials and methods

### Data acquisition

**Figure 1.**
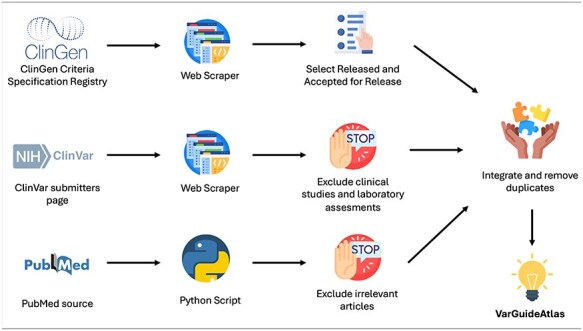
Data acquisition process pipeline.

The development of VarGuideAtlas required a thorough data acquisition process to ensure that existing variant interpretation guidelines were not overlooked. To accomplish this, data were sourced from three primary resources: ClinGen, ClinVar, and PubMed. Figure 1 provides an overview of the process used to obtain interpretation guidelines from each source. A detailed explanation is provided below.

ClinGen (Clinical Genome Resource) is an NIH-funded platform dedicated to defining the clinical relevance of genes and variants for use in precision medicine and research. One of Clingen’s key initiatives is the Criteria Specification Registry (CSpec) [7], which archives variant interpretation guidelines developed by expert panels with extensive domain knowledge for specific genes and diseases.

To extract the interpretation guidelines from the CSpec, we utilized the web scraping capabilities of WebScraper.io, a powerful tool that enables the creation of custom scrapers without coding. This tool allowed for precise and efficient data extraction, streamlining the process while ensuring both accuracy and comprehensiveness.

The CSpec categorizes its guidelines into six maturity levels: *Pilot Rules In Prep, Pilot Rules Submitted, Classification Rules In Prep, Classification Rules Submitted, Accepted for Release*, and *Released*. After obtaining all available guidelines through web scraping, we manually selected only those with a maturity level of *Released* or *Accepted for Release*, as these are the only ones deemed ready by ClinGen for clinical application.

ClinVar is a widely used repository for variant interpretations by clinical experts and laboratories, with over 2 million variant interpretations from more than 2000 contributors [8]. This sheer volume of genomic data underscores its status as one of the most utilized resources in the field. One of its key strengths lies in its ability to provide comprehensive information on the guidelines, clinical studies, and laboratory methodologies used in interpreting each variant, improving traceability, and reproducibility. To build VarGuideAtlas, we extracted, using again WebScraper.io, all clinical guidelines from ClinVar’s submitter page. Then, we manually processed these results to exclude clinical studies and laboratory assessments, thus ensuring that only variant interpretation guidelines were obtained.

**Figure 2.**
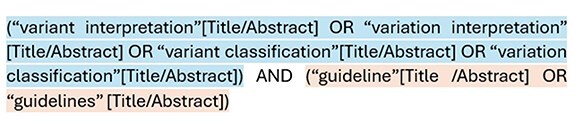
Query structure for the PubMed literature search.

Finally, we look for scientific articles that describe variant interpretation guidelines. To achieve this, we conducted a comprehensive search in PubMed, which contains over 36 million biomedical citations. To streamline this process, we developed a Python script that automates the retrieval of articles from PubMed based on a specific query. For this task, the query depicted in Fig. 2 was used.

The query is structured in two well-differentiated parts. In blue, terms related to variant interpretation, including common synonyms, such as “variation” and “classification,” are included. In orange, we have specified that the term guideline should be introduced. The query was executed in June 2024, and the results were manually processed to exclude irrelevant articles and focus solely on publications that specifically provided guidelines for variant interpretation.

### Data harmonization

The selected guidelines were carefully curated to precisely extract and harmonize their most relevant features. For each guideline, the specific information outlined in Table 1 was gathered.

**Table 1.** Information obtained from each variant interpretation guideline

Attribute	Description	Example
Title	Title of the guideline	A phenotype-enhanced variant classification framework to decrease the burden of missense variants of uncertain significance in Type 1 long QT syndrome
Authors	The person, institution, or laboratory that created the guideline	Bains *et al*.
Variant Type Applicability	Specific variant type to which the guideline applies	Missense variant
Disease Applicability	Specific disease to which the guideline applies	Long QT Syndrome 1
Gene Applicability	Specific gene to which the guideline applies	KCNQ1
URL	Link where the guideline is available	http://dx.doi.org/10.1186/s40246-022-00407-x10.1016/j.hrthm.2021.11.017

A common challenge in medical information is the inconsistency in the terminology used to describe the same concept. To tackle this issue, we used ontologies to limit the number of different instances across three attributes: *Variant Type Applicability, Disease Applicability*, and *Gene Applicability*. Ontologies offer a controlled, structured vocabulary that ensures standardization, providing finite and well-defined terms that enhance consistency, improve communication, and boost interoperability. This standardization is crucial for the comprehensive and consistent characterization of variant interpretation guidelines that VarGuideAtlas aims to deliver.

To identify ontologies suitable for annotating variant types, diseases, and genes, we utilized the Ontology Lookup Service (OLS), a comprehensive repository offering the latest versions of over 200 biomedical ontologies [9]. We manually evaluated the descriptions of these ontologies and chose those that included relevant terms for three key attributes: *Variant Type Applicability, Gene Applicability*, and *Disease Applicability*. This curation process ensured the selection of the most appropriate ontologies for our annotation needs.

As a result, we identified 11 ontologies relevant to characterizing variant interpretation guidelines. Specifically, the SO, EDAM, NGBO, and NCIT ontologies were chosen for *Variant Type Applicability*. The OGG ontology was selected for *Gene Applicability*. Finally, we employed the EFO, MONDO, DOID, ORDO, SNOMED CT, and HP ontologies for Disease Applicability. Table 2 provides a detailed overview of these selected ontologies.

**Table 2.** Ontologies used for standardizing terminology in the cataloged guidelines

Applicability	Acronym	Name	Reference
Variant type	SO	Sequence Ontology	[10]
Variant type	EDAM	Bioinformatics operations, data types, formats, identifiers, and topics	[11]
Variant type	NGBO	Next Generation Biobanking Ontology	[12]
Variant type, Disease	NCIT	NCI Thesaurus OBO Edition	–
Gene	OGG	Ontology of Genes and Genomes	[13]
Disease	EFO	Experimental Factor Ontology	[14]
Disease	MONDO	Mondo Disease Ontology	[15]
Disease	DOID	Human Disease Ontology	[16]
Disease	ORDO	Orphanet Rare Disease Ontology	[17]
Disease	SNOMED CT	Systematized Nomenclature of Medicine—Clinical Terms	[18]
Disease	HP	Human Phenotype Ontology	[19]

Each clinical guideline obtained during the data acquisition stage was manually annotated using the 11 previously selected ontologies. This annotation enabled a precise assessment of their applicability across variant type, gene, and disease dimensions. First, we looked at each guideline to determine its intended clinical context and scope of application. Second, we used the OLS search engine to identify the most suitable ontological terms for describing guideline applicability. The OLS search functionality includes an algorithm that considers various parameters, such as term names, descriptions, and established synonyms. This comprehensive search algorithm facilitated the precise characterization of variant interpretation guidelines, achieving VarGuideAtlas’ primary goal of providing well-annotated, easily accessible guidelines.

### Implementation

**Figure 3.**
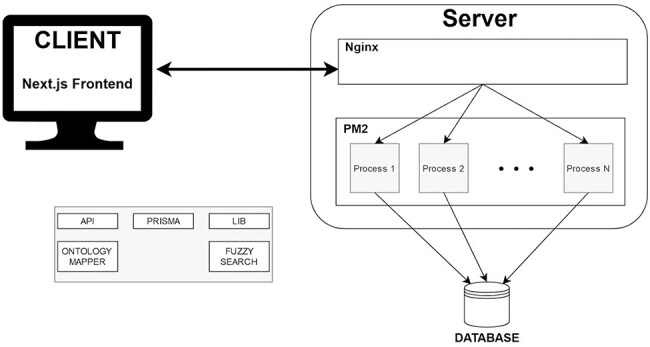
Architecture VarGuideAtlas.

The whole architecture of VarguideAtlas is depicted in Fig. 3. For the database, we chose PostgreSQL (https://www.postgresql.org/), an open-source and powerful object-relational database management system. The database relational model is based on the conceptual model for variant interpretation presented by the same authors in [20]. The choice of PostgreSQL was motivated by its reliability and scalability in data storage and management. Importantly, PostgreSQL’s text search capabilities and the pg_trgm (https://www.postgresql.org/docs/current/pgtrgm.html) extensions were crucial for implementing our fuzzy search functionality. These capabilities allowed us to create specialized indexes to handle fuzzy search operations, making it an ideal choice for our ontology-based search system (see Section User Interface). We chose Prisma (https://www.prisma.io/) as our Object-Relational Mapping library to simplify the interaction with the database. Prisma provides type-safe database access and supports auto-generated migrations. This simplified our initial development process and continues to facilitate schema updates as the project evolves.

We deployed the web-based repository on a Linux server following a horizontally scalable architecture. For application management, we used PM2 (https://pm2.keymetrics.io/), a process manager for Node.js applications. PM2 keeps the application running continuously, enables zero-downtime reloads, and handles logging, monitoring, and clustering. PM2’s cluster mode allows the application to run across multiple CPU cores, dynamically spawning worker processes to handle increased load. We configured the system to automatically scale the number of Node.js instances based on server resources (i.e. the number of available CPUs) and user demand (i.e. the number of server requests from the client), ensuring optimal performance during peak usage. We also employed Nginx (https://nginx.org/) as a reverse proxy server. This configuration enhances security by not exposing our Node.js server directly to the internet and facilitates the implementation of SSL/TLS encryption.

We used JavaScript for client- and server-side programming for development, enabling a unified language across the technological stack. We chose Next.js (https://nextjs.org/) as our full-stack framework due to its server-side rendering, static site generation, and efficient routing. The User Interface was built using TailwindCSS (https://tailwindcss.com/) and NextUI (https://nextui.org/). TailwindCSS, a utility-first CSS framework, streamlined the styling process and ensured a responsive design. Complementing this framework, NextUI provided us with a set of pre-designed UI components and utilities that helped us create a cohesive user interface.

## Results

### Database statistics

Using the automated methods described in the Data acquisition section, we obtained 135 ClinGen guidelines, 515 ClinVar guidelines, and 431 PubMed guidelines, totaling 1081 potential variant interpretation guidelines. ClinVar was the most prolific among these sources, contributing 47.6% of the total guidelines.

**Figure 4.**
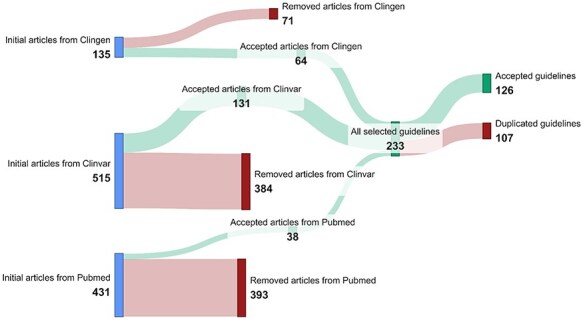
Summary of the data acquisition results.

These guidelines went through a rigorous filtering process. This process resulted in a curated set of 233 guidelines (64 from ClinGen, 131 from ClinVar, and 38 from PubMed), representing a reduction of 78% from the initial findings. Most excluded entries originated from ClinVar and PubMed, primarily consisting of false positives generated by the PubMed queries or records about clinical studies and laboratory assessments rather than interpretation guidelines in ClinVar. Subsequent integration and de-duplication of the remaining results across all sources yielded a final collection of 126 accepted variant interpretation guidelines. The results of the data acquisition process are summarized in Fig. 4.

The 126 guidelines identified were analyzed to obtain the information detailed in Table 1. Here, it is important to highlight that among the 126 curated guidelines, 23 were intended to provide a general framework for variant interpretation, offering alternative approaches to the widely adopted ACMG-AMP guidelines. The remaining 103 guidelines were tailored to address specific variant types, genes, or diseases.

The 126 guidelines also went through the process of ontological characterization of their applicability. The application of the ontologies resulted in 27 unique terms for variant types, 102 terms for genes, and 81 terms for diseases, enabling a granular and systematic classification of the guidelines’ scope and relevance. This ontological and machine-readable annotation of variant interpretation guidelines will allow for efficient identification and retrieval based on specific user needs. Additionally, standardized ontologies enhance interoperability and compatibility with existing knowledge bases and bioinformatics tools, further supporting integration and broader utility.

### User interface

VarGuideAtlas offers a user-friendly web interface with easy access to a curated collection of variant interpretation guidelines. The application is structured into three main pages: Home, Pipeline, and Team.

**Figure 5.**
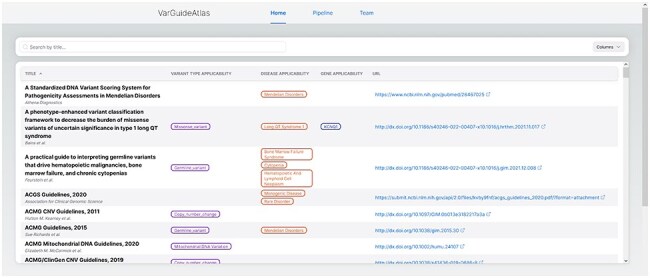
VarGuideAtlas home page. The home page provides a user-friendly interface for searching and exploring the curated collection of variant interpretation guidelines.

The “Home” page (see Fig. 5) serves as the primary interface for discovering and exploring guidelines. It features a comprehensive table presenting the curated clinical guidelines and their associated standardized ontology terms. This table is designed for easy navigation, with sortable columns (i.e. the title and URL columns) and infinite scroll to handle large datasets. When users hover over the ontology terms in the table, the corresponding ontology IDs are displayed, providing additional information and facilitating precise and systematic interpretation across different sources. As mentioned before, this direct integration of standardized terminology enhances interoperability and supports consistent variant interpretation practices.

A key feature of the Home page is its search system, which is integrated with a fuzzy matching algorithm implemented in our database. This advanced search system allows users to quickly identify guidelines based on specific criteria such as variant type, gene, or disease. This search system is designed to enhance the guideline discovery process. It works by creating indexes for each guideline, its name, and the group of associated ontological terms in our system: variant type, gene, and disease. These indexes are optimized for similarity searches, enabling the system to perform a fuzzy search across all three indices simultaneously when a user initiates a query. The system then calculates the corresponding similarities and returns guidelines matching any indexed terms. This approach ensures comprehensive coverage, allowing users to discover relevant guidelines even if their search terms do not match our database’s standardized ontological terms. This flexibility is particularly valuable in the field of medical information, where terminology varies and evolves rapidly.

**Figure 6.**
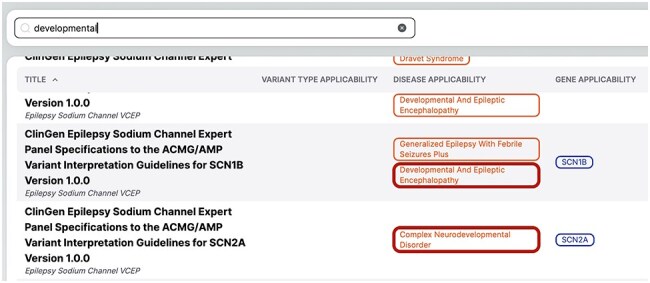
Results of the “developmental” search in VarGuideAtlas.

The fuzzy matching capability is crucial for improving the platform’s usability, as it ensures that users can find relevant guidelines even with partial or imprecise search terms. For example, if a user searches for the term “developmental” in VarGuideAtlas, the system will not only return guidelines explicitly tagged with “developmental” but also those related to derivatives such as “neurodevelopmental.” This feature, illustrated in Fig. 6, highlights the system’s ability to capture a broader scope of relevant guidelines, demonstrating its importance in navigating the complex landscape of medical terminology.

The “Pipeline” page offers a detailed breakdown of the curation process, encompassing the entire workflow from initial data extraction from ClinVar, PubMed, and ClinGen to filtering and de-duplication. This page aims to provide transparency into our methodology. Finally, the “Team” page introduces the authors behind VarGuideAtlas and provides insight into their expertise in genomics and bioinformatics.

## Conclusions and future outlook

### Utility of the database for researchers and clinicians

VarGuideAtlas is a comprehensive repository that centralizes, organizes, and significantly enhances the accessibility and usability of variant interpretation guidelines. By providing a single point of access to a curated collection of 126 guidelines, VarGuideAtlas addresses the challenge of guideline dispersion in the field of genomic medicine. This centralization streamlines the workflow of researchers and clinicians, who can quickly access relevant guidelines without tedious and time-consuming literature searches across several academic repositories. As a result, VarGuideAtlas contributes to more consistent and reliable genomic analyses. This consistency is critical in clinical settings, where it can lead to more accurate diagnosis and treatment decisions. For researchers, it enables better comparison of results across different studies.

The web interface features a robust search system with fuzzy-matching capabilities. This allows users to quickly find relevant guidelines, even when using partial or imprecise search terms For instance, a researcher studying a rare genetic disorder can easily find guidelines tailored to the disease, a particular gene of interest, or both.

Our repository’s emphasis on standardization, interoperability, and ease of use makes it a valuable resource for the bioinformatics community, particularly in the era of precision medicine, where accurate and accessible genomic information is crucial for informed decision-making.

### Uniqueness and relevance of VarGuideAtlas

VarGuideAtlas represents a significant advancement in variant interpretation by centralizing and improving access to a vast number of variant interpretation guidelines. This repository addresses the longstanding issue of guideline dispersion, which has hindered experts’ ability to efficiently locate and apply the most appropriate guidelines for their specific needs.

One of the key strengths of VarGuideAtlas lies in its use of ontologies to standardize the variant type, gene, and disease applicability of the guidelines. This approach allows for a precise characterization of each guideline and facilitates interoperability with other bioinformatics tools and databases. By providing a machine-readable, ontology-driven framework, VarGuideAtlas significantly improves the efficiency of identifying and retrieving relevant guidelines, ensuring that users can navigate the existing guidelines with greater ease and accuracy.

Integrating a fuzzy matching algorithm into the search system further enhances the usability of VarGuideAtlas. The ability to perform similarity searches across the clinical guidelines provided by our resource allows users to discover guidelines that might otherwise be missed due to discrepancies in terminology. We consider this feature to be particularly relevant in this domain, where terminology varies across different sources and over time.

To our knowledge, no other resource that focuses on providing a centralized repository specifically for interpretation guidelines exists. This repository, together with its standardized ontology-based annotation of guidelines and advanced search capabilities, positions VarGuideAtlas as a tool that fills a critical gap in the current ecosystem of genomic resources.

### Limitations of VarGuideAtlas

Although VarGuideAtlas represents a significant advancement in managing variant interpretation guidelines, two limitations warrant consideration. First, the repository relies on manual curation. While ensuring that the included guidelines are highly accurate and relevant, this process is inherently time-consuming. Furthermore, the manual curation process may cause delays between the publication of new guidelines or updates to existing ones and their inclusion in VarGuideAtlas, temporarily limiting access to the most current information. As existing clinical guidelines grow over time, this manual process could hinder the repository’s ability to scale efficiently and provide updated and comprehensive coverage.

Secondly, VarGuideAtlas’s current focus on English-language resources risks overlooking valuable guidelines published in other languages, limiting the repository’s global utility. This limitation becomes increasingly significant when considering the genetic diversity of different populations worldwide. Each population has unique genetic characteristics, which may necessitate specialized interpretation guidelines. As our understanding of population-specific genetic variations improves, guidelines will most likely become more geographically specific. This trend toward regionalizing genetic interpretation guidelines raises the possibility that clinical guidelines will be published in local languages, making them inaccessible to a system primarily focused on English-language resources.

These challenges highlight the need for ongoing development and refinement of VarGuideAtlas to ensure it continues to serve as a comprehensive and up-to-date resource for the genomics community worldwide.

### Future updates of the database

VarGuideAtlas is committed to continuous improvement and expansion to meet the evolving needs of the genomics community. Our future development plans focus on several key areas that will enhance the repository’s functionality, efficiency, and user experience.

A primary focus will be the automation of the curation process. We aim to develop and implement automated systems for guideline extraction, filtering, and initial classification. These will significantly improve the efficiency of VarGuideAtlas, allowing it to keep pace with the rapidly expanding field of genomics while maintaining the high quality of curated content.

This new automated system will obtain not only ontology terms but also ontology descriptions. This addition aims to improve our search capabilities by incorporating an advanced Natural Language Processing system that matches free-text clinical descriptions to available ontology descriptions. The user will input the free-text clinical description via an extended UI on our website. This system will address the common challenge of clinicians using non-standard or descriptive terminology (e.g. “additional number of fingers on both hands” for “polydactyly”), making the repository more accessible to users who are unfamiliar with formal clinical nomenclature. In tandem with the abovementioned proposals, more general plans include exploring and implementing advanced machine learning algorithms for automated guideline classification and annotation, improved fuzzy matching and search capabilities, and intelligent recommendation systems to suggest relevant guidelines based on user queries and profiles.

Another important feature to introduce in VarGuideAtlas is the automatic update of the information it provides. New variant interpretation guidelines will appear reported in the three resources considered in this work. Besides, ClinGen’s CSpec is constantly updated with improved versions of the same guidelines. Therefore, to ensure that VarGuideAtlas provides the most updated knowledge, we plan to provide at least once a year a new version of VarGuideAtlas. This will be facilitated by the high levels of automatization we plan to achieve with the above-mentioned improvements.

Recognizing the importance of global accessibility, we are committed to expanding our language coverage, ensuring that valuable guidelines published in languages other than English are not overlooked. By broadening our linguistic scope, we aim to make VarGuideAtlas a truly global resource that reflects the genetic diversity of populations worldwide.

To enhance interoperability and facilitate integration with other bioinformatics tools, we will develop a comprehensive API. This will allow programmatic access to the repository, enabling researchers and developers to incorporate VarGuideAtlas data directly into their workflows and applications. Additionally, we will implement a robust version control system to track guideline updates and revisions over time, providing users with a clear history of changes and ensuring transparency.

Finally, to foster collaboration within the genomics community, we plan to introduce features such as user-contributed annotations and comments on guidelines, a platform for experts to discuss and debate guideline applications, and integration with existing scientific and social networks. These collaborative features will help create a dynamic, community-driven resource that evolves with the field.

## Data Availability

All data are available upon reasonable request.
